# Nursing Services Delivery Theory: an open system approach

**DOI:** 10.1111/j.1365-2648.2010.05449.x

**Published:** 2010-12

**Authors:** Raquel M Meyer, Linda L O’Brien-Pallas

**Affiliations:** Raquel M. Meyer PhD RN Nursing Early Career Researcher Ontario Ministry of Health & Long-Term Care, and Assistant Professor (CLTA) Lawrence Bloomberg Faculty of Nursing, University of TorontoOntario, Canada; Linda L. O’Brien-Pallas PhD RN FCAHS Professor & CHSRF/CIHR Chair in Nursing Human Resources Lawrence Bloomberg Faculty of Nursing, University of TorontoOntario, Canada

**Keywords:** nursing management, Nursing Services Delivery Theory, open system approach, organization structure, quality of care, staffing, work organization

## Abstract

meyer r.m. & o’brien-pallas l.l. (2010)Nursing services delivery theory: an open system approach. *Journal of Advanced Nursing***66**(12), 2828–2838.

**Aim:**

This paper is a discussion of the derivation of the Nursing Services Delivery Theory from the application of open system theory to large-scale organizations.

**Background:**

The underlying mechanisms by which staffing indicators influence outcomes remain under-theorized and unmeasured, resulting in a ‘black box’ that masks the nature and organization of nursing work. Theory linking nursing work, staffing, work environments, and outcomes in different settings is urgently needed to inform management decisions about the allocation of nurse staffing resources in organizations.

**Data sources:**

A search of CINAHL and Business Source Premier for the years 1980–2008 was conducted using the following terms: theory, models, organization, organizational structure, management, administration, nursing units, and nursing. Seminal works were included.

**Discussion:**

The healthcare organization is conceptualized as an open system characterized by energy transformation, a dynamic steady state, negative entropy, event cycles, negative feedback, differentiation, integration and coordination, and equifinality. The Nursing Services Delivery Theory proposes that input, throughput, and output factors interact dynamically to influence the global work demands placed on nursing work groups at the point of care in production subsystems.

**Implications for nursing:**

The Nursing Services Delivery Theory can be applied to varied settings, cultures, and countries and supports the study of multi-level phenomena and cross-level effects.

**Conclusion:**

The Nursing Services Delivery Theory gives a relational structure for reconciling disparate streams of research related to nursing work, staffing, and work environments. The theory can guide future research and the management of nursing services in large-scale healthcare organizations.

**What is already known about this topic**

- Because the delivery of nursing services has typically been investigated using hospital-level staffing indicators, the underlying mechanisms by which nursing work influences outcomes remain under-theorized and unmeasured.
- Large-scale organizations can be conceptualized as open systems composed of interacting subsystems that selectively import and transform energic inputs from the external environment to produce services and products.

**What this paper adds**

- The healthcare organization is conceptualized as an open system characterized by energy transformation, a dynamic steady state, negative entropy, event cycles, negative feedback, differentiation, integration and coordination, and equifinality.
- This theory situates the work of nursing in the production subsystems of the organization and explicates the division and coordination of nursing work.
- The theory gives a relational structure that reconciles how nursing work, staffing, and work environment variables contribute to the global work demands placed on nurses at the point of care.

**Implications for practice and/or policy**

- Future research can be guided by this theory to examine how variations in inputs, throughputs, and organizational characteristics result in optimal outputs related to nursing services delivery.
- Managers can use this theory as an overarching framework to manage the key components conceptualized to influence the delivery of nursing services at the point of care in organizations.

## Introduction

In many countries, the sustainability and quality of nursing services are threatened by global shortages of healthcare professionals (International Council of Nurses 2006). As one component of a multifaceted response to this crisis, policy and decision-makers have prioritized the nursing practice environment and organizational performance as key areas for intervention (International Council of Nurses 2006). Nursing services are generally contracted through an employment relationship. To recruit, retain, and deploy scarce nursing human resources effectively and to produce quality and cost-effective care, the associations between organizational structures, human resource management policies and the goals, resources, context, and outcomes of nurses’ work need to be understood. A challenge in nursing health services research has been the need for a unifying theory to conceptualize and examine the delivery of nursing services (Edwardson 2007).

In the conceptual model for nursing and health policy, Russell and Fawcett (2005) identified four levels of focus: (i) nursing practice processes; (ii) administrative practices for nursing service (or healthcare) delivery subsystems; (iii) healthcare system administrative practices; and (iv) world health administrative practices. The Nursing Services Delivery Theory (NSDT) addresses the second level of phenomena in this model by examining the effectiveness and efficiency of administrative practices for nursing service delivery subsystems. Using the strategy of theory derivation, the NSDT gives a theoretical understanding of the nature of an organization, situates the work of nurses in the organizational context, and integrates the design and organization of nursing work. In this study, we present a description of the derivation of the NSDT from the application of Open System Theory to large-scale organizations and the structural and conceptual elements of the NSDT. Examples from the empirical literature are used to illustrate the relational structure the NSDT describes among nursing work, work environment, and staffing variables. Further implications of the theory are discussed.

## Background

Nursing health services research is characterized by a growing need for a coherent theoretical framework that combines clinical, organizational, financial, and outcome variables from a nursing perspective (Edwardson 2007). Nurse staffing studies often apply traditional nurse staffing indicators to give crude estimates of the amount of nursing resources available for care. However, by virtue of their simplicity, nurse staffing indicators also de-contextualize care. Conceptually, hours per patient day (HPPD) assume a standard time per occupied bed, whereas nurse–patient ratios are based on average nurse capacity (O’Brien-Pallas *et al.* 2005). Workload measurement systems quantify patients’ requirements for nursing care as the sum of the times of the tasks required or as the amount of time required relative to standard patients (Thibault *et al.* 1990). In the community, numbers of visits reflect standard times allotted per home visit. These types of nurse staffing indicators inadequately consider factors known to influence variability in nursing work, namely the characteristics of care recipients and nursing teams, and factors related to the care delivery environment (O’Brien-Pallas *et al.* 1997).

Although staffing research examining large administrative data sets in the United States of America (USA), Canada, and the United Kingdom has identified associations between key nurse staffing indicators and patient outcomes at the organizational level (Lankshear *et al.* 2005), these types of secondary analyses do not measure the actual work performed by nurses (Clarke 2006). Thus, the underlying mechanisms by which staffing indicators influence outcomes remain under-theorized and unmeasured, resulting in a ‘black box’ that masks the nature and organization of nursing work. Although large database studies allow for comparisons across organizations, evidence on which to re-organize and improve nursing services to varied clinical populations at the point of care is lacking (Clarke 2006). Specific theory and evidence linking staffing practices and outcomes in different settings are urgently needed to inform management decisions about the allocation of nurse staffing resources in organizations.

A review of funded nursing health services research in the USA identified that conceptual frameworks were often used in isolation by researchers (Edwardson 2007). Donabedian’s (1980) Healthcare Organization and Delivery Model is one of the most frequently used frameworks to examine nursing performance (Hall 2004, Edwardson 2007). Conceptual frameworks of nursing care based on Donabedian’s (1980) formulation for the assessment of care quality typically organize patient, nurse, work, work environment, and outcome variables according to structure, process, and outcome (e.g. Irvine *et al.* 1998, Cho 2001). However, because Donabedian (1980) was focused on an approach for assessing the quality of medical care, rather than on system design and organization, the fundamental questions of ‘What is an organization?’ and ‘What is nursing work?’ remain unanswered. Rationales for including variables in a structure–process–outcome framework have tended to rely on empirical findings, rather than a theoretical understanding of the nature of an organization or the delivery of nursing services. In addition, because linear relationships are frequently assumed between structure, process, and outcome variables, the dynamic interactions between variables are often neglected (Mitchell *et al.* 1998).

Building on a rich tradition of systems thinking in clinical (Holden 2005) and nursing management, the NSDT addresses many of these challenges to nurse staffing and nursing work research. In particular, Jelinek (1967) described a Patient Care System Model composed of personnel types and physical facilities as inputs; organizational and environmental factors as throughput; and patient care, patient satisfaction, and personnel satisfaction as outputs. Subsequently, the interrelationships among nursing complexity, medical complexity, nurse characteristics, environmental complexity, and outcomes were tested in a systems model in community and hospital settings to investigate the factors that cause patients or clients with very similar medical conditions to have different nurse resource requirements (O’Brien-Pallas *et al.* 1997, 2001, 2002, 2004, Meyer *et al.* 2009). In these models, inputs consisted of the characteristics of patients or clients, nurses, and the system and system behaviours; throughput involved the nursing care delivery subsystem, where nursing interventions are performed and its environmental complexity; and outputs involved outcomes for patients or clients, nurses, and the system. Mark *et al.* (1996) also applied structural contingency theory, a subset of Open System Theory, to the evaluation of nursing system outcomes. Key variables included environment (e.g. organizational size, skill mix), technology (e.g. stability of patient acuity, diversity of patient conditions), structure (e.g. degree of centralization), and effectiveness (e.g. patient and administrative outcomes). The basic premise was that to perform effectively and to produce quality outcomes, an organization must structure its nursing units to complement the environment and technology.

The NSDT complements and extends the scope of previous systems models in nursing by theorizing the nature of an organization, locating the work of nursing at the work group level in an organizational suprasystem, and explicating the division and coordination of nursing work. By viewing the healthcare organization through the lens of objectivism, the work of nurses is assumed to exist as an objective, external reality with identifiable and measurable characteristics.

The theory derivation was guided by these questions: What is the nature of an organization? How do healthcare organizations produce nursing services? How do management structures contribute to the delivery of nursing services? According to Walker and Avant (2005), theory derivation is an iterative and creative process that involves: (i) becoming very familiar with the level of theory development in the field and evaluating existing theories; (ii) reading widely both in and outside the field of study to make creative associations between distinct fields of study; (iii) choosing a parent theory for the derivation; (iv) identifying which content and structural elements of the parent theory will be used; and (v) recasting these elements for the phenomenon of interest.

## Data sources

Building on our pre-existing knowledge, literature from the nursing, healthcare, and management fields was examined. A search of CINAHL and Business Source Premier was conducted using combinations of the following terms: theory, models, organization, organizational structure, management, administration, nursing units, and nursing. The search was limited to English language, peer-reviewed publications or books published between 1980 and 2008. Seminal works were also included. Documents with a major focus on theory related to work performance and management in organizations were reviewed.

## Discussion

Katz and Kahn’s (1978)*The Social Psychology of Organizations*, based on Open System Theory, was selected as the parent theory because it addressed the questions guiding the derivation in a comprehensive manner and facilitated new insights and connections about research in the areas of nursing work, staffing, and work environments. Selected structural and conceptual elements of the parent theory were redefined. Specifically, the open system characteristics of organizations and the five functional subsystem types, which are the fundamental defining characteristic of social systems (Katz & Kahn 1978), were recast in the NSDT with an emphasis on the dynamics and mechanisms of production subsystems.

### Open System Theory

The theoretical foundation of the NSDT is Open System Theory as applied to large-scale organizations by Katz and Kahn (1978). In their view, an organization constitutes an energic input–output system. An organization depends on its supporting environment for continued inputs to ensure its sustainability and processes these inputs through the recurring and patterned activities and interactions of individuals to yield outputs. An organization is therefore essentially a social system. As such, an organization and its subsystems strive to achieve a dynamic steady state whereby regularities in energy flow preserve the character of the system and disturbances prompt system adaptation (Katz & Kahn 1978). To survive, an organization needs to counteract entropy, which is an inevitable process of disorder and dissolution caused by loss of inputs or by inability to transform energies. An open system must acquire negentropy (i.e. negative entropy), usually through some form of storage capacity, to ensure its continued existence (Katz & Kahn 1978). For organizations, negentropy can involve renewing inputs, storing energy, creating slack resources, or maximizing imported energy relative to exported energy (Galbraith 1974, Katz & Kahn 1978). Organizations can also counteract entropy by adapting system functioning in response to informational signals and feedback from the environment. Characteristics of open systems and their application to large-scale organizations and to the NSDT are presented in Table 1.

**Table 1:** Open system concepts and their corollary in the Nursing Services Delivery Theory

Concept	Definition (Katz & Kahn 1978)	Application to large-scale organization (Katz & Kahn 1978)	Application to the Nursing Services Delivery Theory
Inputs	The inflow of energy and information from the external environment renews the system	Energic inputs may include people, materials, or resources from other organizations. Informational inputs include negative feedback or signals about the external environment	People – e.g. staff, care recipientsMaterials – e.g. suppliesResources – e.g. funding Information – e.g. labour market conditions
Throughput	Energies inside the system are transformed by reorganizing the inputs	Reorganization may entail processing of materials, generation of products, or provision of services	Services – e.g. nursing interventions
Output	Product must be exported to the external environment	Outputs may consist of materials, products, or services	Service outputs – e.g. patient volumes
Systems as cycles of events	The process of exchanging and transforming energy must renew the system thus creating a repeated series of activities	Renewal may be generated by system output or by its own activities	Outputs – e. g., revenuesActivities – e.g. accreditation criteria achieved
Negative feedback	Internal information about system functioning is a corrective device used to adjust energy intake and expenditure	Subsystem feedback about operational functioning is used to keep the organization on target	Negative feedback – e.g. organizational performance indicators

Open System Theory recognizes the hierarchical nature of entities, whereby each level of the organization comprises a ‘subsystem’ of interrelated parts. In large-scale organizations, the transformation of energy (i.e. throughput) occurs in production subsystems that divide the labour to accomplish tasks (Katz & Kahn 1978). The function of the production subsystem is to transform energy to meet task requirements and to optimize task accomplishment via technical proficiency (Katz & Kahn 1978). The underpinning mechanism is the division of labour that determines the structure and work flow in the production subsystem. Subdividing the work creates breaks in work flow. Organizations address this challenge by integrating work processes across roles and subunits using coordination devices (Katz & Kahn 1978). As an organization differentiates, additional integration and coordination are required to unify system functioning (Katz & Kahn 1978). Thus, the size, complexity, and coordination demands of an organization increase as its subsystems multiply and specialize in function.

**Figure 1:**
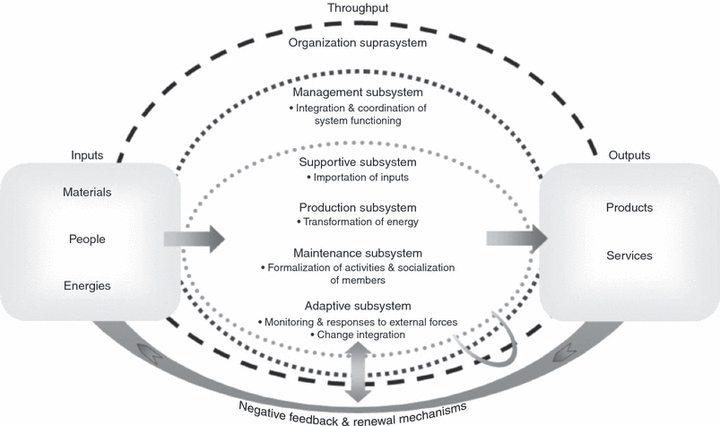
Simplified representation of the organization as an open system based on Katz & Kahn (Meyer 2010, reproduced with permission).

The production subsystems interact dynamically with the supportive, maintenance, and adaptive subsystems of the broader organization (Figure 1). These subsystems import people, materials, and energies through transactions at the organizational boundaries; balance internal work structures relative to human inputs by formalizing activities and socializing and rewarding members; and deal with problems of adjustment to external forces by recommending and incorporating change (Katz & Kahn 1978). Overall organizational functioning and adjustment to external demands are coordinated and integrated by the management subsystem, which crosscuts and directs all subsystems and negotiates conflict across hierarchical levels (Katz & Kahn 1978). In terms of functioning, the production, supportive, maintenance, adaptive, and management subsystems do not operate in isolation, but rather are interdependent and interact dynamically as part of a greater, complex whole.

As an open system, the organization adapts its functioning in response to negative feedback and external informational signals through a series of iterative adjustments that allow the system to evolve while maintaining its character (Katz & Kahn 1978). Although Figure 1 is a simplified representation of the organization as an open system, the phenomenon is neither uni-dimensional nor static. Large-scale organizations typically consist of multiple interacting subsystems (e.g. multiple production subsystems by specialty, hierarchically layered management subsystems). The principle of equifinality states that an open system can achieve its end state from various initial conditions and through differing trajectories (Katz & Kahn 1978). This suggests that there is no single way for an organization to be structured or to achieve positive outcomes.

### Nursing Services Delivery Theory

**Figure 2:**
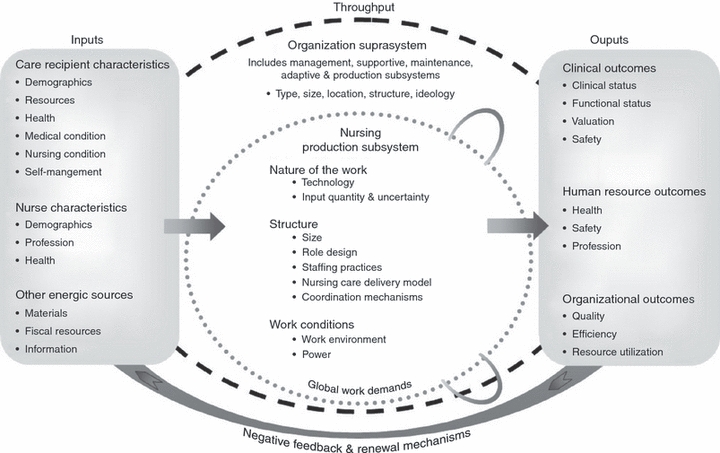
The Nursing Services Delivery Theory.

The NSDT applies Open System Theory to nursing work in large-scale healthcare organizations (Figure 2). With respect to system structure, the NSDT identifies that care is delivered by nurses clustered in work groups that are nested in a department or programme in the larger organization. Inpatient units in a hospital or nursing teams in home healthcare are examples of production subsystems. These work groups transform energic inputs to deliver nursing services and to yield outputs. Imported inputs consist of care recipients, staff, material and fiscal resources and information, which are subsequently transformed in a nursing production subsystem through the work performed, its structure, and its internal work conditions.

Distal outputs include clinical, human resource, and organizational outcomes. These energic outputs give feedback and reactivate the system in a cyclical manner because positive outcomes in each of these domains ensure that members of the community continue to use the organization’s services, staff are retained to give the services, and the organization’s accreditation and funding are sustained. With respect to nurse staffing, entropy may be counteracted in several ways. Examples of negentropy include: (i) renewal of inputs by retaining or hiring nurses; (ii) storing energy by using buffer inventories of nursing capacity (e.g. float pools, agency nursing); (iii) creation of slack resources by loosening performance targets to reduce the number of exceptions (e.g. longer lengths of stay), by increasing fiscal resources (e.g. greater nursing HPPD), or by extending lead times (e.g. richer staffing ratios); and (iv) more efficient use of imported energy relative to exported energy by intensifying nursing workload to increase volumes (e.g. lower staffing ratios).

In Open System Theory, each system and its subsystems adapt to internal and external demands and feedback. Demands external to the healthcare organization encompass environmental factors (e.g. labour market, legislation, population characteristics). Healthcare organizations continuously adapt system functioning in response to feedback and informational signals to counteract entropy. For example, aligning organizational policies to meet performance targets set by external agencies exemplifies the dynamic interaction between the organization and its external environment.

At the point of care, each nursing production subsystem also adapts to and interacts reciprocally with the other organizational subsystems. The management, supportive, maintenance, and adaptive subsystems coordinate and allocate the inflow of energic inputs and establish the structures necessary for the completion, evaluation, and renewal of nursing work in production subsystems. Internal demands of nursing production subsystems relate to the nature of the work performed, structures arising from the division of nursing labour, and the work conditions at the point of care. Negative feedback includes organizational performance indicators (e.g. longer than expected length of stay or time on programme).

The dynamic interdependence among subsystems, the organization, and the external environment is illustrated using the example of emergency department overcrowding and one of its proposed solutions, the introduction of nurse practitioners. In response to pressures to reduce crowding (i.e. external demand), subsystems would recommend and implement the proposed solution (i.e. adaptive function); hire the nurse practitioners (i.e. supportive function); formalize policies to enable the work of nurse practitioners (i.e. maintenance function); and integrate these changes across subsystem, role, and hierarchical boundaries to ensure stakeholder buy-in and to monitor performance (i.e. management function). The emergency department (i.e. nursing production subsystem) would re-divide the labour to accommodate the new role and the work performed (i.e. internal demands). By altering the staffing mix (i.e. inputs), service capacity (i.e. throughput) is increased, leading to reduced overcrowding and increased consumer satisfaction (i.e. organizational and clinical outcomes; e.g. Carter & Chochinov 2007). In turn, because nurse practitioners typically engage in primary care and health promotion, unnecessary readmissions to the emergency department could be offset in the future (i.e. feedback cycle).

By considering the various inputs and throughputs that influence nursing service delivery and outputs, the NSDT proposes that nursing work in a given production subsystem is not performed in isolation; rather, nursing work in production subsystems is dynamically interdependent with the other subsystems and the organization suprasystem that interact with the external environment. There is no single way for an organization, or for nursing production subsystems, to deliver nursing services effectively. The NSDT emphasizes that a confluence of factors determines the global work demands in the nursing production subsystem.

### Components of the Nursing Services Delivery Theory

As shown in Figure 2, the delivery of nursing services in production subsystems occurs inside the environment structured by the organization suprasystem, and is dependent on the inflow of inputs, which include care recipients, nurses, materials, and other energies.

*Characteristics of the organizational suprasystem* include organizational type, size, location, structure, and ideology. Type of organization can vary by healthcare sector (e.g. acute, community or long-term care), academic affiliation, or by funding source. Location may refer to geography (e.g. rural, urban) or dispersion (e.g. multi-site organization, catchment areas). Depending on the purpose of the inquiry, organizational size can be measured as the quantity of personnel, physical capacity, volume of inputs or outputs, or discretionary resources of an organization (Kimberly 1976). Organizational structure results from trade-offs between the differentiation of work by function (e.g. nursing, pharmacy) and the integration of work processes by programme (e.g. cardiology, trauma; Charnes & Tewksbury 1993). This gives rise to a continuum of functional, matrix, and programme organizational forms (Charnes & Tewksbury 1993). Ideology refers to the common norms and values held by the majority of organizational members about expected member behaviours and the appropriateness of organizational activities and functions (Katz & Kahn 1978).

*Care recipient characteristics* include demographics, health status, resources, medical condition, and nursing condition. Examples of demographic variables are age, gender, language, and ethnicity. Health status includes the physiological and psychosocial health states of the person. Resources available to care recipients can be considered in terms of material support (e.g. healthcare coverage) and social support (e.g. informal caregivers). Medical condition encompasses the number and types of medical diagnoses and co-morbidities and severity. Nursing condition refers to the healthcare needs of recipients that generate the demand for nursing services in terms of complexity (e.g. number and types of nursing diagnoses) or intensity (e.g. workload). Self-care management involves the pre-existing knowledge, health behaviours, and symptom management of care recipients and their informal caregivers about the underlying health conditions.

*Nurse characteristics* consist of demographic, professional, and health factors. Demographics include age and gender. Profession reflects occupational factors such as licensure, education, clinical expertise, experience, and employment status. Health entails the physiological and psychosocial health states of the nurse.

*Other energic sources* include materials, fiscal resources, and information. Materials consist of equipment and supplies. Fiscal resources refer to the budget allocated to a production subsystem. Information can include, but is not limited to, organizational trends and policies, new technologies, and feedback that the production subsystem imports from other organizational subsystems.

*Throughput* consists of several factors. Nursing work is performed in the production subsystem. Key factors influencing the delivery of nursing services in the production subsystem entail the nature of the work, its structure, and its environment. Technology refers to the work performed by nurses. Nursing work may be conceptualized as independent and collaborative interventions that encompass ‘any treatment, based upon clinical judgment and knowledge, which a nurse performs to enhance patient/client outcomes’ (McCloskey & Bulechek 2000, p. 3). In terms of the nursing work performed, technology refers to task uncertainty (i.e. degree to which cause and effect are analysable), instability (i.e. the degree to which moment to moment changes in care recipient status occur), and variability (i.e. diversity of number of different components; Overton *et al.* 1977). The extent to which tasks are interdependent (Thompson 1967) and time-constrained (Adler 1995) is an additional dimension of work performance. Temporal dimensions of nurses’ work (e.g. duration, temporality, timing, tempo) may also be considered (Jones 2001). Uncertainty, instability, variability, interdependence, and timing of nursing work and interventions are amenable to measurement. Quantity and uncertainty of inputs also influence nursing work in the production subsystem. Input quantity may be reflected by volumes of care recipient admissions, visits, procedures, or patients; by number of staff or nursing HPPD; or by fiscal resources. Input uncertainty is determined by the number and probability of choices or alternatives in a given situation (Argote 1982). With respect to care recipients, uncertainty is reflected by diversity in the health conditions and care needs of the population served and the number of exceptional cases encountered. For example, patient flow to a haemodialysis unit is more predictable in terms of admission rates and patterns, service times, sequencing, and health conditions, compared with an emergency department. Uncertainty in staffing inputs is exemplified by, but not limited to, nursing skill mix, team composition, the proportion of full-time staff, and the use of overtime and agency staff to meet demand. Uncertainty in material inputs entails changes in the allocation of fiscal and material resources.

The structure of the nursing production subsystem arises from the division and coordination of nursing work through management subsystem decisions about size, role design, staffing practices, nursing care delivery models, and coordination mechanisms. Size refers to the capacity to produce services (e.g. numbers of beds or available home visits). Role design assigns responsibility for particular tasks to distinct job descriptions. Staffing practices refers to the ways in which care activities and responsibilities are divided among nurses at a micro-level based on care recipients (e.g. nurse–patient ratios, workload scores), staff characteristics (e.g. experience levels), or management practices (e.g. length and scheduling of shifts). Nursing care delivery models (e.g. team, primary or total care models) describe how nursing work is divided and coordinated at the work group level. Coordination entails mechanisms to standardize skills, work processes (e.g. clinical pathways), outcomes, or communication methods (e.g. electronic health record; March & Simon 1958, Venkatraman 1994). Feedback is another coordination device that fosters the exchange of information in an adaptive and reciprocal manner (Gittell 2002). Feedback can occur through direct supervision, boundary spanning roles (e.g. case managers), or teamwork (Gittell 2002). Work conditions internal to the production subsystem encompass the various physical, cognitive, psycho-social, and professional dimensions of the work environment that influence professional practice (Kristensen 1999, Registered Nurses’ Association of Ontario 2008). In the systems approach, power is typically conceptualized as a resource. Empowered work environments are those in which all employees can access opportunities to learn and grow and can obtain the information, support, and resources necessary for the job (Kanter 1977).

The *outputs* in NSDT reflect key outcomes of nurses’ work and work environments. Clinical outcomes sensitive to nursing care can be grouped along four dimensions (White *et al.* 2005). Clinical status outcomes involve the control or management of symptoms (White *et al.* 2005) and the prevention of complications (Irvine *et al.* 1998). Functional status outcomes encompass the physical and psychosocial functioning and self-care abilities of the individual (White *et al.* 2005). Valuation refers to care recipients’ perceptions and appraisals of nursing care and care results (e.g. satisfaction; White *et al.* 2005). Safety outcomes include adverse events and complications (White *et al.* 2005). Human resource outcomes are related to staff members’ physical and mental well-being, safety (e.g. injuries, violence), and profession (e.g. autonomous practice, work satisfaction). System outcomes incorporate evaluations of service quality (e.g. rates of adverse events), efficiency (e.g. target volumes, length on service), and resource utilization (e.g. staffing stability, costs).

### Application

Examples from the empirical literature illustrate the relational structure of the NSDT using inputs, throughputs, and outputs that are integral to nursing health services research. In a first example, Doran *et al.* (2006) explored the relationships between patient characteristics (i.e. inputs), nursing interventions (i.e. throughput), and clinical outcomes (i.e. outputs). Patient functional and cognitive status and depression (i.e. health), but not age (i.e. demographics), were associated with the types of nursing interventions performed (i.e. technology). Nursing interventions in turn partially mediated the relationship between functional status at baseline and at discharge, suggesting that other variables, such as patients’ pre-existing health conditions and the work of other healthcare professionals, may be also influencing outcome achievement (Doran *et al.* 2006).

A second example illustrates the use of buffer inventories to respond to unpredictable staffing needs. Float pool and agency nurses (i.e. inputs) have the potential to lower organizational costs (i.e. resource utilization), but may be detrimental to clinical outcomes under certain conditions. In a study of an intensive care unit, after controlling for the characteristics of patients (e.g. demographics, medical condition), patients receiving care from a higher ratio of pool and agency nurses to permanently assigned nurses (i.e. input uncertainty) were at significantly greater risk for blood stream infections (i.e. clinical status; Robert *et al.* 2000). These authors surmised that agency and float pool staff may receive less training with respect to central venous catheter care and may be less familiar with team functioning and unit practices.

A third example highlights the potential for inter-professional practice to improve care delivery. For example, in a study of joint replacement surgery, workgroups with high levels of teamwork were associated with improved clinical and organizational outcomes (Gittell 2004). The work performed was concurrent and iterative (i.e. interdependent), involved multidisciplinary roles (i.e. variable), and was delivered under declining lengths of stay (i.e. time constrained). The throughputs consisted of teamwork (i.e. coordination mechanism), patient volumes (i.e. input quantity), and a specialized patient population (i.e. low input uncertainty). Outputs included patient satisfaction with care quality (i.e. valuation) and length of stay (i.e. efficiency). After controlling for patient demographics (i.e. patient characteristics), volume enhanced the positive effects of specialization on teamwork and on outcomes. Teamwork in turn mediated the effect of specialization on improved outcomes, suggesting that the benefits of specialization are in part achieved through high levels of teamwork (Gittell 2004).

In a final example, nursing work environments remain a key priority among healthcare employers, particularly for staff recruitment and retention in the context of nursing shortages. A study of new nurse graduates (i.e. inputs) revealed that those who experienced greater employee-job fit (i.e. throughput) were more likely to report improved human resource outcomes (i.e. outputs; Cho *et al.* 2006). When nurses with less than two and a half years experience (i.e. nurse characteristics) had access to opportunity, information, support, and resources (i.e. power), they were more likely to perceive greater fit in terms of workload, control, rewards, community, fairness, and values (i.e. work environment), and in turn they reported higher work engagement and consequently less burnout and greater organizational commitment (i.e. nurse health and profession outcomes). These examples highlight the capability of the overarching framework and the conceptual underpinnings of the NSDT to support theoretical connections among distinct streams of nursing services research related to nursing work, work environments, and staffing variables.

## Implications for nursing

The NSDT explains the contributions of the organizational suprasystem and its subsystems to the global work demands placed on nurses in production subsystems. To avoid a black box approach to investigating the work of nurses, the actual work performed (e.g. technology) by nurses at the point of care, and not merely the structures or work conditions, should be measured. The NSDT also integrates the nested nature of organizational phenomena, thereby encouraging the study of multiple levels of phenomena and the examination of cross-level effects and interactions. Because the NSDT offers an abstracted view of the phenomena and is broad in scope, its components cannot be tested comprehensively or directly in any single study.

Future research can be guided by this theory to examine how variations in inputs, throughputs, and organizational characteristics result in optimal outputs related to nursing services delivery. Empirical indicators need to be chosen carefully to reflect the concepts in the NSDT. The components of the NSDT are interactive and dynamic, not static. Depending on the specific hypotheses to be tested, the empirical indicators used to represent NSDT concepts may serve as independent or dependent variables to given equations in the analytical models (Jelinek 1967). As conceptualized, the input, throughput, and output components of the NSDT are likely to be relevant across countries, cultures, and settings because of the open system premise. However, the measurement of phenomena (i.e. selection of empirical indicators) may be tailored to specific countries, cultures, or settings. The fields of organizational design and organizational behaviour can assist in building hypotheses, assuming that the principles of open systems are upheld, to examine why and how variations in inputs, throughputs, and suprasystem characteristics result in optimal outputs across nursing services delivery production subsystems.

The NSDT can also be used to manage the factors influencing nursing services delivery in organizations. Accumulated and synthesized evidence is needed to explain the conditions under which the delivery of nursing services in large-scale healthcare organizations influences clinical, human resource, and organizational outcomes.

## Conclusion

Nursing health services research has often been criticized for being atheoretical. Investigation of a wide variety of nurse staffing and work environment indicators has contributed to a fragmented understanding of nursing services delivery and nurses’ work. By placing these studies in the relational structure of the NSDT, a theoretical basis is given for reconciling disparate streams of research. The NSDT can facilitate the identification of unstudied gaps and the selection of conceptually meaningful variables for future research. The NSDT also offers managers new insights with which to prioritize and evaluate concurrent organizational initiatives directed at increasing nursing service efficiency, effectiveness, and sustainability. The NSDT thus offers an overarching theory for examining and managing the key concepts theorized to influence the delivery of nursing services at the point of care in large-scale healthcare organizations.
